# AcceSS and Equity in Transplantation (ASSET) New Zealand: Protocol for population-wide data linkage platform to investigate equity in access to kidney failure health services in New Zealand

**DOI:** 10.1371/journal.pone.0273371

**Published:** 2022-08-25

**Authors:** Rachel B. Cutting, Angela C. Webster, Nicholas B. Cross, Heather Dunckley, Ben Beaglehole, Ian Dittmer, John Irvine, Curtis Walker, Merryn Jones, Melanie Wyld, Patrick J. Kelly, Kate Wyburn, Nicole L. De La Mata

**Affiliations:** 1 Sydney School of Public Health, Faculty of Medicine and Health, University of Sydney, Camperdown, NSW, Australia; 2 National Health and Medical Research Council Clinical Trials Centre, University of Sydney, Camperdown, NSW, Australia; 3 Westmead Applied Research Centre, Westmead Hospital, Westmead, NSW, Australia; 4 Department of Nephrology, Canterbury District Health Board, Christchurch Hospital, Christchurch, New Zealand; 5 New Zealand Transplantation and Immunogenetics Laboratory, New Zealand Blood Service, Auckland, New Zealand; 6 Department of Psychological Medicine, University of Otago, Christchurch, New Zealand; 7 Auckland City Hospital, Auckland District Hospital Board, Auckland, New Zealand; 8 Internal Medicine, Medical Council of New Zealand, Mid Central District Health Board, Wellington, New Zealand; 9 Renal Services, Ballantyne House, Hawke’s Bay District Health Board, Hastings, New Zealand; 10 Renal Unit, Royal Prince Alfred Hospital, Sydney Local Health District, Camperdown, NSW, Australia; Medical University of Gdansk, POLAND

## Abstract

**Background:**

Kidney transplantation is considered the ideal treatment for most people with kidney failure, conferring both survival and quality of life advantages, and is more cost effective than dialysis. Yet, current health systems may serve some people better than others, creating inequities in access to kidney failure treatments and health outcomes. AcceSS and Equity in Transplantation (ASSET) investigators aim to create a linked data platform to facilitate research enquiry into equity of health service delivery for people with kidney failure in New Zealand.

**Methods:**

The New Zealand Ministry of Health will use patients’ National Health Index (NHI) numbers to deterministically link individual records held in existing registry and administrative health databases in New Zealand to create the data platform. The initial data linkage will include a study population of incident patients captured in the Australia and New Zealand Dialysis and Transplant Registry (ANZDATA), New Zealand Blood Service Database and the Australia and New Zealand Living Kidney Donor Registry (ANZLKD) from 2006 to 2019 and their linked health data. Health data sources will include National Non-Admitted Patient Collection Data, National Minimum Dataset, Cancer Registry, Programme for the Integration of Mental Health Data (PRIMHD), Pharmaceutical Claims Database and Mortality Collection Database. Initial exemplar studies include 1) kidney waitlist dynamics and pathway to transplantation; 2) impact of mental illness on accessing kidney waitlist and transplantation; 3) health service use of living donors following donation.

**Conclusion:**

The AcceSS and Equity in Transplantation (ASSET) linked data platform will provide opportunity for population-based health services research to examine equity in health care delivery and health outcomes in New Zealand. It also offers potential to inform future service planning by identifying where improvements can be made in the current health system to promote equity in access to health services for those in New Zealand.

## Introduction

Chronic kidney disease (CKD) is a public health concern and a burden to health systems globally [1, 2]. The global prevalence of CKD was estimated at 9.1% of the population in 2017 and accounted for 1.2 million deaths that year [1]. In 2019, CKD was ranked the 6th leading cause of death in the United States and the 7th leading cause of death in Australia [3]. Between 2009–2019, CKD rose from the 8th to 7th leading cause of death in New Zealand [3, 4]. The most severe stage of CKD (stage five) is classified as kidney failure [4]. Treatment options for kidney failure are dialysis (peritoneal or haemodialysis), kidney transplantation or supportive care (also called conservative care or non-dialytic care) [4]. Kidney transplantation is considered the preferred treatment as it reduces morbidity and mortality and is more cost-effective [5–9]. As demand for kidney organs outweighs supply, those assessed as suitable for transplantation remain active on the waitlist until a kidney donor becomes available for transplant [4, 10, 11].

**Fig 1:**
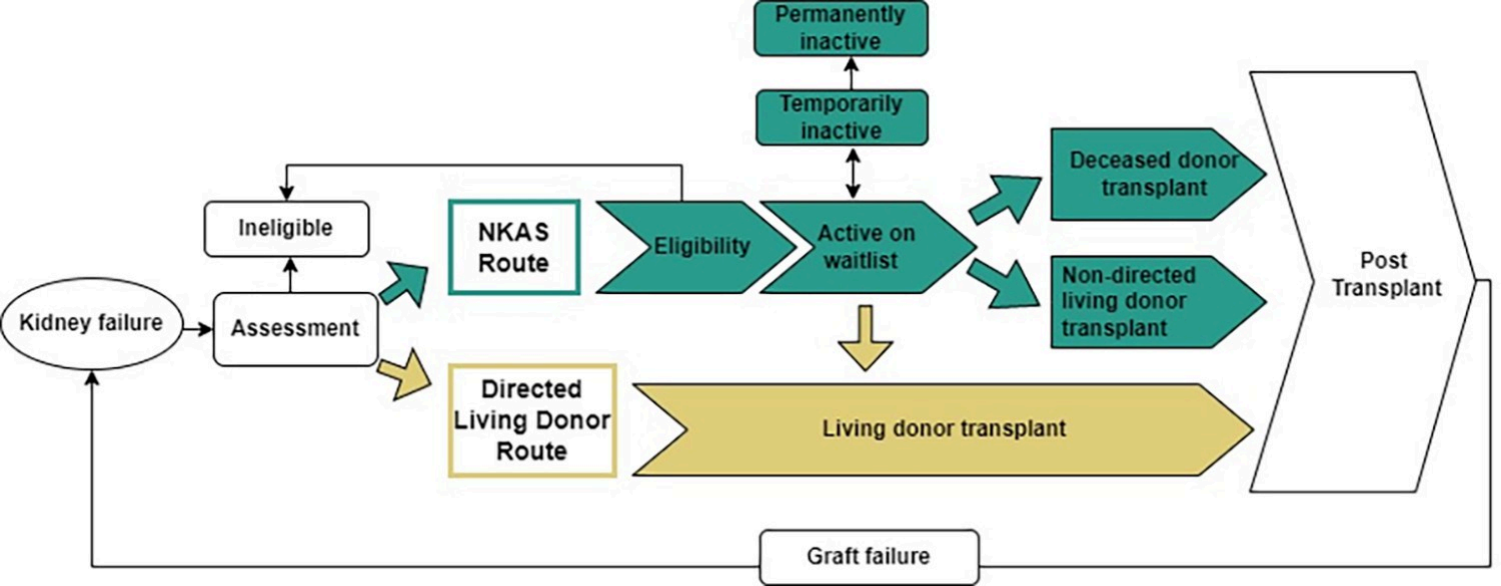
The kidney transplant pathway in New Zealand via the NKAS and the directed living donor route. Patients may be involved in one route or both routes at times.

The kidney transplant pathway is complex and comprises two different routes (Fig 1). One is via the National Kidney Allocation Scheme (NKAS), which requires waitlisting for a deceased or non-directed living donor (altruistic living donors without an intended recipient) [10]. The other is through directed living donation, where a person with an enduring relationship from within the recipients circle of family and friends donates directly to the recipient [12, 13]. The two routes to transplantation are not mutually exclusive. An eligible recipient may receive a deceased donor kidney while a directed live donor is in assessment. Or, once a directed live donor is fully assessed, suitable and available to donate, the recipient can be removed from the deceased donor waiting list and the live donor transplantation proceeds.

New Zealand has one centralised, national kidney waitlist for deceased and non-directed living donor kidneys allocated using the NKAS [4, 10]. Waitlist eligibility requirements are detailed in the Clinical Guidelines for Organ Transplantation from Deceased Donors governed by the Transplantation Society of Australia and New Zealand (TSANZ) [10]. Eligibility includes: low risk of surgical complications, heart attack, or stroke; the absence of active malignancies or infection and an estimated survival probability of greater than 80% at five years post-transplant [4, 10]. These requirements limit access to the waitlist for those with multimorbidities who may not gain survival or quality of life benefits from transplantation, and on utilitarian grounds, due to the limited supply of organs for transplantation. The NKAS uses an allocation algorithm to allocate deceased donor kidneys to eligible people on the kidney waitlist. The algorithm makes best use of available organs for longevity and provides fair access to transplantation for those on the waitlist. The algorithm is reviewed annually and is periodically adjusted, which subsequently may increase equitable access to certain groups of people.

The NKAS route includes referral for eligibility assessments prior to pre-waitlist tests, placement on the active waitlist, remaining well on the waitlist, allocation of a kidney from a suitable donor, surgery and postoperative recovery and ongoing healthcare to maintain graft function [10]. Along the pathway, some people will commence assessment, yet never actually become active (for some, because they will be eligible for a living donor transplant and have an available live donor), others will be accepted and active on the waitlist but subsequently be temporarily or permanently removed from the waitlist, and some will die before receiving a transplant. Being temporarily inactive for an extended period of time, or experiencing multiple transitions between temporarily inactive and active, results in missed opportunities for transplantation and longer time spent on dialysis, which is associated with increased risk of clinical deterioration and death. Graft failure may occur following transplantation, resulting in the possibility of repeating the assessment and waitlist process.

The directed living donor route includes those who have been offered a kidney from a known donor, usually genetically or emotionally related [10, 13]. An individual with kidney failure can be on the NKAS route and transition to work-up for a directed living donor transplant. Although, patients with assessed, eligible and available living donors are no longer eligible for a deceased donor kidney at that point and proceed to living donor transplantation. Directed living donor transplantation can occur at any stage prior to transplant via NKAS with approximately 40% of living donor transplants occurring as the first treatment for kidney failure in New Zealand, most of which are directed [10, 14]. Further, if the potential living donor does not proceed with donation, patients can transition from directed living donor route to the NKAS route.

Equitable access to the kidney waitlist and subsequent transplantation is challenging. There are known disparities internationally based on ethnicity, geographic location and sex [7, 15–19]. In Australia, indigenous people are less likely than non-indigenous to be waitlisted or transplanted (SHR 0.46 (95% confidence interval (CI) 0.38–0.55)) [7]. In New Zealand, Māori and Pasifika transplant crude rates were lower than average compared to non-Māori and non-Pasifika between the years 2014–2019 [20]. Though Māori and Pasifika are more likely to have comorbidity which may contribute to decreased transplant access, it is not clear if other factors such as socioeconomic status and location are also at play [20]. Additionally, systematic bias in delivery of health services may contribute further to inequity for Māori [21, 22]. Any inequity in access to kidney transplantation by ethnicity in New Zealand has not been systematically appraised, though it likely occurs, given Māori and Pasifika experience lower access to healthcare services [23, 24].

Equitable access to the kidney waitlist and transplantation in New Zealand may be disproportionate based on sex and geographical proximity to health services. Evidence in Australia has shown between the years 1993–2012 adolescents and children with kidney failure living in remote and regional areas were 35% less likely to receive a pre-emptive living kidney transplant compared to those in metropolitan areas (adjusted OR 0.65, (95% CI 0.45–1.0; *p*  =  0.05)) [18]. Living in regional Australia is associated with reduced likelihood of being waitlisted compared to residing in urban settings (SHR 0.88 (95% CI 0.81–0.95)) [7]. In Denmark, regional variation in incidence of dialysis and transplantation treatment between 2001–2006 showed high rates in the cities Copenhagen and Aarhus where major nephrology centres are located (standardised for ethnic origin, age and sex: (Copenhagen ((164 p.m.p.) Aarhus (156 p.m.p.) compared to other regions (120 p.m.p.) (P < 0.0001)) [19]. However, this could be influenced by other factors such as differences in health behaviour and treatment centre practices [19]. Furthermore, in the United States, women have lower access to the waitlist compared to men (HR 0.89 (95% CI, 0.89 to 0.90, fully adjusted)) [25]. Also, disparity in access to transplantation for women compared to men widens with increasing age [15, 16]. In Australia, adult females were less likely than males to be waitlisted or transplanted between the years 2006–2015 (SHR 0.85 (95% CI 0.80–0.91)) [7]. ASSET, a population data-linkage platform, will be used to explore if similar sex differences and geographic variations occur and impact access to transplantation in New Zealand.

Data linkage to administrative health databases and registry-held data offers a means of identifying intervention points to address equity in health service delivery to allow individuals opportunity to reach their full health potential. As per the World Health Organisation and the New Zealand Ministry of Health’s definition, the authors of this study define health equity as the absence of not only avoidable or remediable differences, but also unfair and unjust differences in access to kidney failure services for people in New Zealand [21, 26]. Health equity in New Zealand also represents an obligation to Māori under the Te Tiriti o Waitangi (the Treaty of Waitangi), stating the importance and responsibility of the Crown to protect, promote and meet the health needs and health aspirations of Māori [21]. Data linkage, the process of matching records in different databases from the same individual, can provide a more detailed picture of individuals’ interactions with the health system. This can be performed deterministically; using exact matching of information, such as email addresses, phone numbers and healthcare numbers; or probabilistically; identifying records that most likely belong to the same patient based on personal identifiers such as date of birth, sex, residence, and date of death. Data linkage offers advantages by being more cost-effective and efficient than observational cohort studies. The wealth of health data can address multiple research questions and includes all people in a population, as opposed to a sample, with complete lifelong follow up. New Zealand is an ideal setting for data linkage. NHI numbers are included within health and other databases and therefore capture individuals’ contact with health services. NHI numbers also allow for deterministic linkage which are less likely to result in incorrect links than probabilistic linkage.

## Materials and methods

ASSET will create meaningful and impactful partnerships with researchers and health professionals, including investigators with Māori whakapapa and/or experience in engagement with Māori communities and applying Kaupapa Māori research methodologies. In a collaborative approach, ASSET investigators will use a health services perspective and a comprehensive data platform to examine equity in kidney failure health service delivery in New Zealand.

### Aims

ASSET investigators aim to create a comprehensive and enduring data-linkage platform to enable collaborative health services research in New Zealand. This protocol outlines the processes of establishing the ASSET linked data platform and provides some exemplar studies to initially investigate equity gaps in access to transplantation in New Zealand.

### Study design

ASSET is a population wide data-linkage platform of existing national registry and administrative health databases in New Zealand. ASSET facilitates a systems-based approach to investigate equity in access to kidney failure health services and provides opportunity for further health services enquiry.

### Population

**Fig 2:**
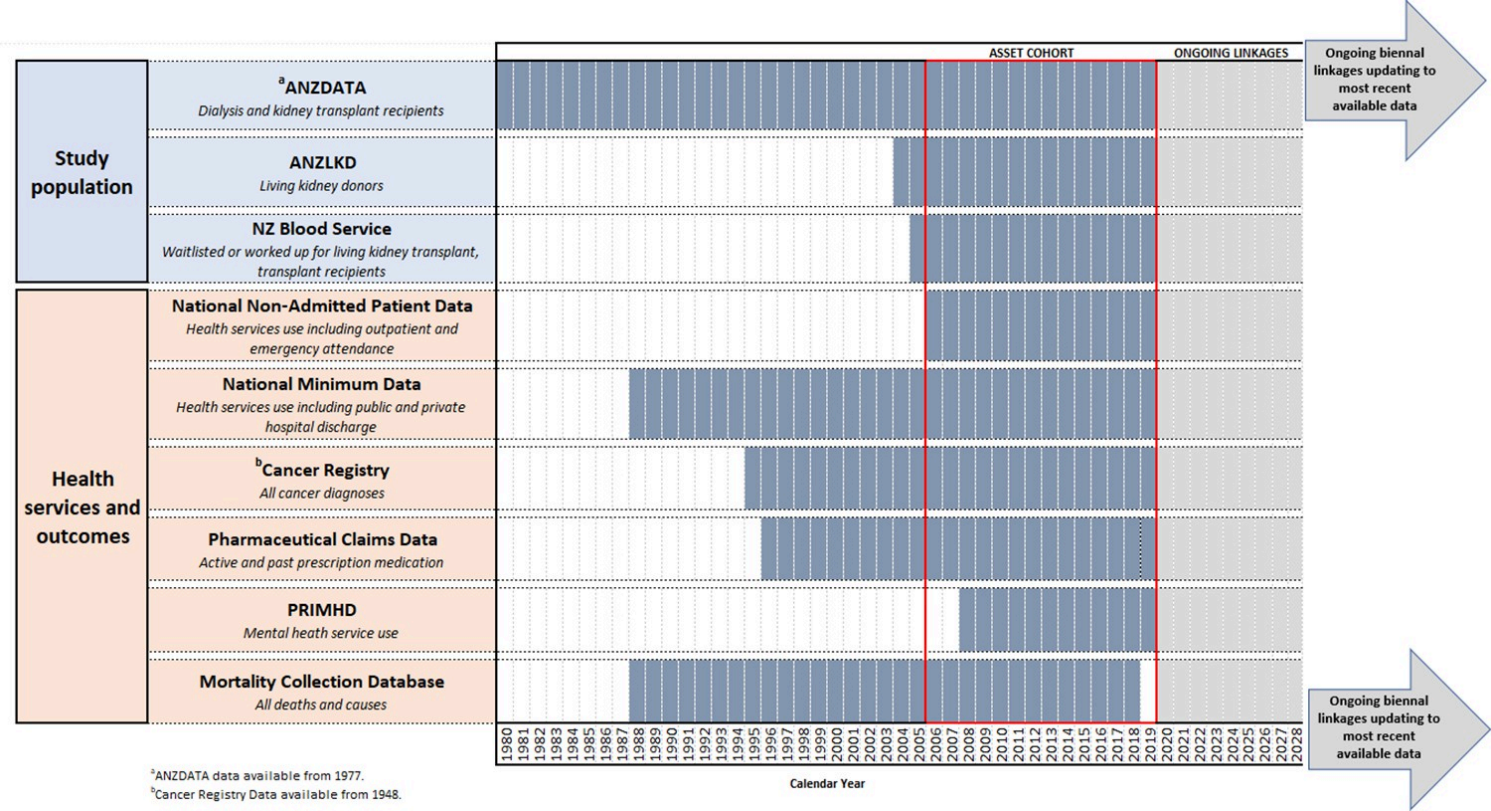
Calendar years of available data from each data source included in the initial data linkage to create ASSET data platform. Study population databases include ANZDATA (Australia and New Zealand Dialysis and Transplant Registry), ANZLKD (Australia and New Zealand Living Kidney Donor Registry) and NZ Blood Service (New Zealand Blood Service Database). Health outcomes databases include National Non-Admitted Patient Data, National Minimum Data, Cancer Registry, Pharmaceutical Claims Data, PRIMHD (Programme for the Integration of Mental Health Data) and Mortality Collection Database. ASSET data platform will be relinked biennially to include most recent available data and any additional databases for ongoing health services research.

ASSET consists of all people receiving treatment for kidney failure in New Zealand. This includes all people receiving dialysis, ever waitlisted or worked up for a living kidney transplant, all kidney transplant recipients, including those who received pre-emptive transplants and all living kidney donors. Our initial data linkage will include incident patients in the study population between 1 Jan 2006 and 31 December 2019 (Fig 2). We anticipate our study population will encompass approximately 15,000 patients receiving treatment for kidney failure, or ever waitlisted or worked up for a living kidney transplant and 1,500 living kidney donors.

### Data sources for initial data linkage

#### Study population

*Population with kidney failure receiving kidney replacement therapy*. Australia and New Zealand Dialysis and Transplant Registry (ANZDATA) records data on all persons receiving dialysis and those who undergo transplantation within Australia and New Zealand from 1977 onwards. This data source will define those with kidney failure receiving dialysis and kidney transplant recipients in New Zealand between 1980–2019. New Zealand Blood Service Database records all persons in New Zealand waitlisted for kidney transplant, (including waitlist status changes over time) or worked up for living kidney transplant, and those who received transplants, including pre-emptive, between 2005–2019.

*Living kidney donor population*. Australia and New Zealand Living Kidney Donor Registry (ANZLKD) records data on all living donors in Australia and New Zealand between 2004–2019. Only New Zealand living donors will be included in ASSET.

#### Health services and outcomes

*Hospital admissions and emergency attendance*. National Non-Admitted Patient Collection Data and National Minimum Dataset collects and records patterns of health service use, including hospital admissions, outpatient and emergency department contact events from 1988–2019. These data will provide health service usage that will inform reasons for being temporarily inactive or permanently inactive, post-transplant outcomes, reasons for death, comorbidities and living donor health resource use.

*Cancer diagnoses*. Cancer Registry in New Zealand collects and records data on all cancer diagnoses between 1995–2019. This will identify cancers in the study population to inform analyses.

*Mental health data*. Programme for the Integration of Mental Health Data (PRIMHD) records service activity and outcomes for all health consumers who received treatment from public sector secondary care and non-governmental organisation mental health and addiction services between 2008–2019. This database will capture those with severe mental illness, as patients with mild or moderate mental illness are largely cared for in primary health services.

*Prescription medications*. Pharmaceutical Claims Database records dispensing of subsidised medications (the vast majority of all medication dispensed in New Zealand) between 1996–2019. This will identify comorbid conditions requiring medication, such as cardiovascular disease. It will also be used to define a group with moderate mental illness and severe mental illness not captured in PRIMHD.

*Deaths*. Mortality Collection Database records all deaths and their causes using International Classification of Diseases (ICD) between 1988–2018. This will provide date and cause of death for our study population.

### Initial exemplar projects

The ASSET linked data platform is designed to address multifaceted health services enquiry, with a focus on health equity. Three initial exemplar projects are listed below to provide context and feasibility of intended research using ASSET. Any analyses and findings produced from ASSET projects will be made available in published peer-reviewed journal articles, technical reports and other dissemination output upon completion. Project specific data will be available from the corresponding author for any reasonable request.

1. Kidney waitlist dynamics (ASSET WL): Evaluating equity in the journey to kidney transplant, considering allocation algorithm changes, including being considered for the waitlist, being active on the waitlist, transplantation (including directed and non-directed living donor transplant and pre-emptive transplant), and post-transplant outcomes.
2. Mental health and kidney failure (ASSET MH): Assessing whether people with mental illness and kidney failure have fair access to transplantation, and similar outcomes, i.e., survival and graft function, compared to those without mental illness.
3. Living donor health service use (ASSET LD): Examining the health consequences of living kidney donation by investigating health service use after donation in living donors compared to other New Zealanders.

### Factors and their impact on access to transplantation

The factors considered in our initial analysis and their impact on access to transplantation and post-transplant outcomes include health service factors, patient characteristics and demography. Health service factors include proximity to transplant centres (distance between location of residence and treatment centre) and District Health Board (DHB) service locations. Patient characteristics include multimorbidity (e.g. mental health, diabetes, cardiovascular disease, cancer etc) and frailty. Demography includes age, sex, ethnicity and socioeconomic status (by location of residence).

### Health service factors

#### Location of residence, proximity to transplant centres and District Health Board service locations

ASSET data platform can be used to assess if living far from kidney failure treatment centres and living in certain DHB’s influence access to transplantation. In New Zealand, transplant centres are located in Auckland, Wellington and Christchurch [20]. For those living further from urban areas, the time, logistics, and costs associated with travelling to transplant centres may negatively impact access [27].

There are twenty DHB’s in New Zealand, each providing services for their population and, at times, other DHB populations [28]. Kidney failure health services are managed by transplanting DHBs (transplantation services) Auckland, Capital and Coast, and Canterbury [20]. Referring DHB’s (dialysis services) are Northland, Waitemata, Counties Manukau, Taranaki, Waikato, MidCentral, Hawkes Bay and Southern [20]. Referring DHB’s without comprehensive services (depending on other DHB’s for some elements of dialysis services) are Tairawhiti, Lakes, Bay of Plenty, Whanganui, Hutt, Nelson Marlborough, Wairarapa, West Coast and South Canterbury [20]. Some people may access services across three DHB locations, whereas others, such as those living in Capital and Coast, can access comprehensive services without needing services from other DHB’s. The impact of living in certain DHB’s is perhaps more pronounced than geography in New Zealand. For example, areas within Canterbury DHB are remote, yet Canterbury contains transplant services and has the highest transplantation rate [20]. Whereas areas within Bay of Plenty are less remote, yet transplant services are provided further away in Auckland. Therefore, ASSET provides scope to explore if living in certain DHB’s impact access to transplantation.

### Patient characteristics

#### Multimorbidity and frailty

Kidney failure rarely occurs without the presence of other chronic diseases. Diabetes, hypertension and cardiovascular disease are associated with developing CKD [29].

Multimorbidity, the presence of two or more chronic conditions, contributes to complexity in care requiring access to many health services. For those with mental illness and kidney failure, navigating multiple health services and managing complex health needs is likely to create difficulties with accessing and maintaining engagement in health care. ASSET investigators will consider the impact of mental illness on access to transplantation and post-transplant outcomes, by comparing data to those who have no identified mental illness. Multimorbidity is also associated with increased risk of frailty [30]. Frailty is defined as an increased vulnerability to stressors with impaired ability to return to homeostasis after a stressor event [31]. ASSET generates granular information to investigate the impact of multi-morbidity burden, including those with mental illness, on access to transplantation.

### Demography

#### Age

The impact of age on accessing transplantation and post-transplant outcomes in New Zealand can be examined using ASSET. Globally, the incidence rate of treated kidney failure increases with advancing age [32, 33]. The current clinical guidelines by TSANZ states that age in itself is not an excluding factor for waitlisting, but the presence of co-morbidities and decreased survival probability with older age would result in the majority of the elderly population being ineligible [10]. ASSET can be used as a resource to explore the impact of age and associated comorbidities and to better understand if exclusion from the waitlist is justified.

#### Sex and gender

There remains disparity in accessing kidney transplantation by sex. In Australia, women are less likely to be waitlisted or receive kidney transplants than men [32]. ASSET provides opportunity to investigate if service delivery is different by sex in New Zealand, and any influence on waitlist transitions and post-transplant outcomes. These data report sex as binary: male or female. Gender, a social construct, is not recorded. This means those who are part of the LGBTIQ+ community and are gender nonconforming are not distinguished. We acknowledge the importance of including genderqueer in health research yet are constrained by limitations of the data.

#### Ethnicity

In New Zealand, rates of kidney transplantation differ by ethnicity. Māori and Pasifika experience higher incidence rates of kidney failure yet lower transplant rates compared to non-Māori and non-Pasifika [34]. Reasons for this are not well established but may relate to geographic location, multimorbidity, socioeconomic status, patient preferences, health literacy, health practitioners’ attitudes, and lack of shared decision making and culturally-appropriate education [6, 20, 35, 36]. ASSET enables deeper understanding of the impact of ethnicity on access to transplantation and post-transplant outcomes.

#### Socio-economic status (by location of residence)

ASSET investigators will use patient’s statistical area codes to incorporate the NZDep, a measure of deprivation index, to understand the impact of socioeconomic status on access to transplantation. NZDep estimates relative socioeconomic deprivation from census data including variables such as income, employment, crime, housing, health, education and access to services such as internet, supermarkets, service stations and schools [37]. Higher levels of socioeconomic deprivation (SED) is associated with poorer health, yet, a recent study in New Zealand found no difference in overall survival post deceased donor transplantation for those living in high SED areas in New Zealand compared to those living in low SED areas (HR 1.5, SE 0.47 (95% CI: 0.82–2.76;p = 0.19)) [38]. Interestingly, higher SED areas had a 53% greater transplant rate than lower SED in New Zealand (Odds ratio [OR]1.53 (95% CI: 1.33–1.76;p< 0.00005)) [38]. ASSET provides opportunity to examine the impact of socioeconomic status throughout the entire transplant pathway as well as post-transplant outcomes by using NZDep from location of residence within the linked data.

### Intersectional advantage and disadvantage

Factors such as age, ethnicity, multimorbidity and socioeconomic status (location of residence) do not exist independently and may potentiate each other to result in greater advantage or disadvantage. For example, living in particular DHB’s in conjunction with multimorbidity may have a clustering effect on barriers in accessing transplantation for Māori and Pasifika people. ASSET has the capacity to explore and compare the occurrence of intersecting factors and whether these effects compound in reducing access to transplantation.

### Data linkage

**Fig 3:**
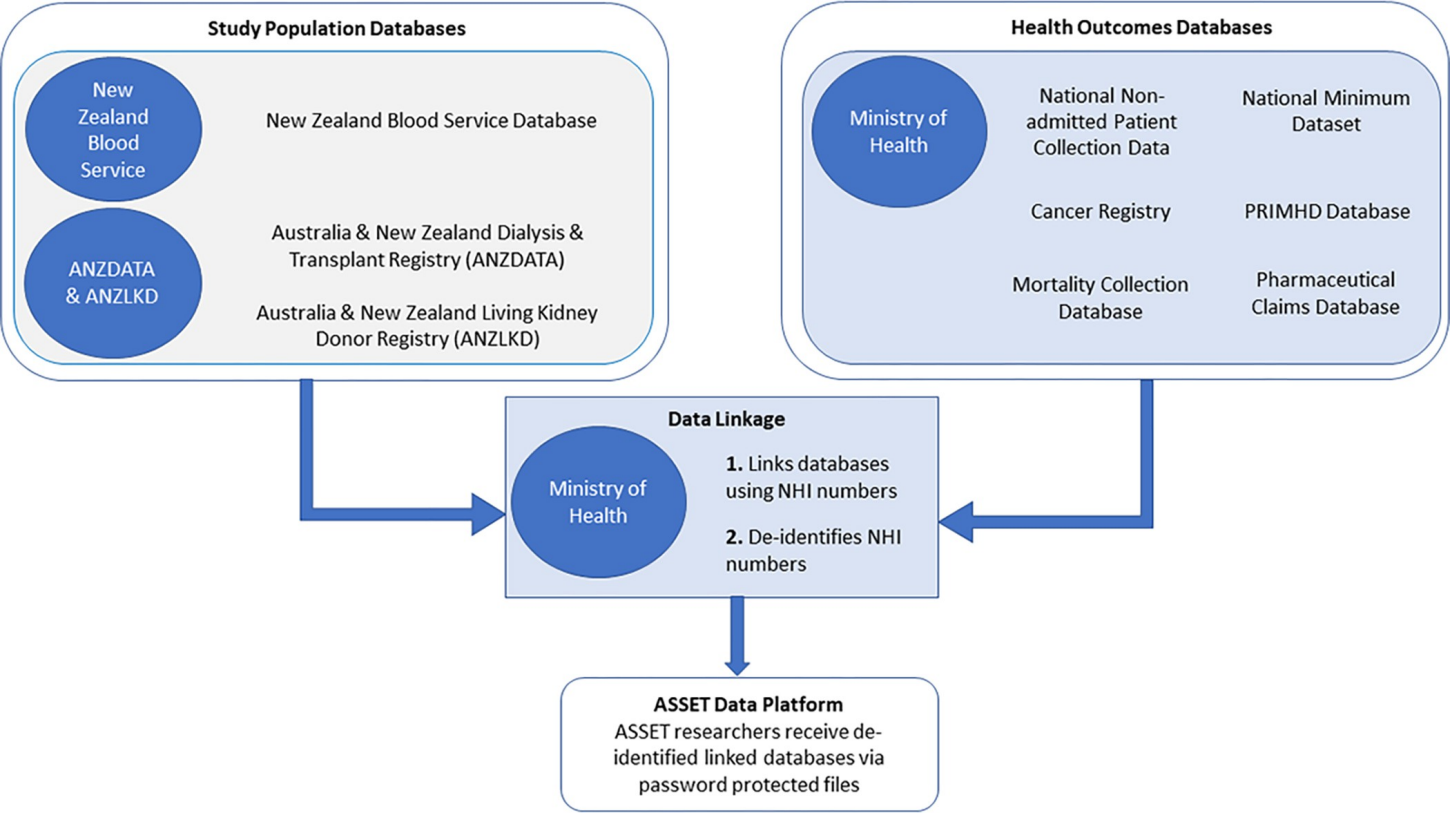
Process of data linkage by the New Zealand Ministry of Health. Linking study population and health services and outcomes databases to create the ASSET linked data platform.

Data linkage will be performed by the New Zealand Ministry of Health. All databases will be deterministically linked using NHI numbers (Fig 3). Those missing NHI numbers will be linked using probabilistic data, matching on personal identifiers including name, sex and date of birth. It is anticipated that only 1% of the study population will be missing NHI numbers. Deterministic linkage using NHI numbers is particularly important, as care for kidney failure patients occurs wholly in the public system and will be captured by health databases.

For additional confidentiality precautions, ANZDATA and ANZLKD will send all identifying NHI numbers directly to the New Zealand Ministry of Health for linkage and content data to ASSET researchers at the University of Sydney. The New Zealand Blood Service will send both NHI numbers and content data to the New Zealand Ministry of Health for linkage. Once all databases are received by the New Zealand Ministry of Health, data linkage will be undertaken followed by the encryption of all NHI numbers to de-identify the data. The de-identified data will be sent via password protected files to the researchers at the University of Sydney and stored on the University’s secure server. The risk of re-identification of patients is minimal.

### Data processing

ASSET will be a sustainable data resource with re-linkage occurring every two years (biennial) to ensure the platform is updated with current data. The biennial re-linkage will also provide capacity for inclusion of additional databases for further health services enquiry. Potential new databases currently under discussion include justice health, vaccination records and incorporating further health information of living donors sourced directly from New Zealand transplant units. ASSET investigators are also developing a way to capture data of people with kidney failure who do not undergo active treatment and instead transition directly to conservative care.

### Ethical considerations and governance framework

ASSET was approved by the University of Sydney Human Research Ethics Committee HREC 2020/871 on March 26, 2021. The Health and Disability Ethics Committee, New Zealand determined ASSET was out of scope for ethics review due to the use of de-identified data. Approval was therefore not required. Best privacy preserving practices will be upheld and all data accessed by researchers will be in de-identified format.

#### Data oversight and governance roles

**Fig 4:**
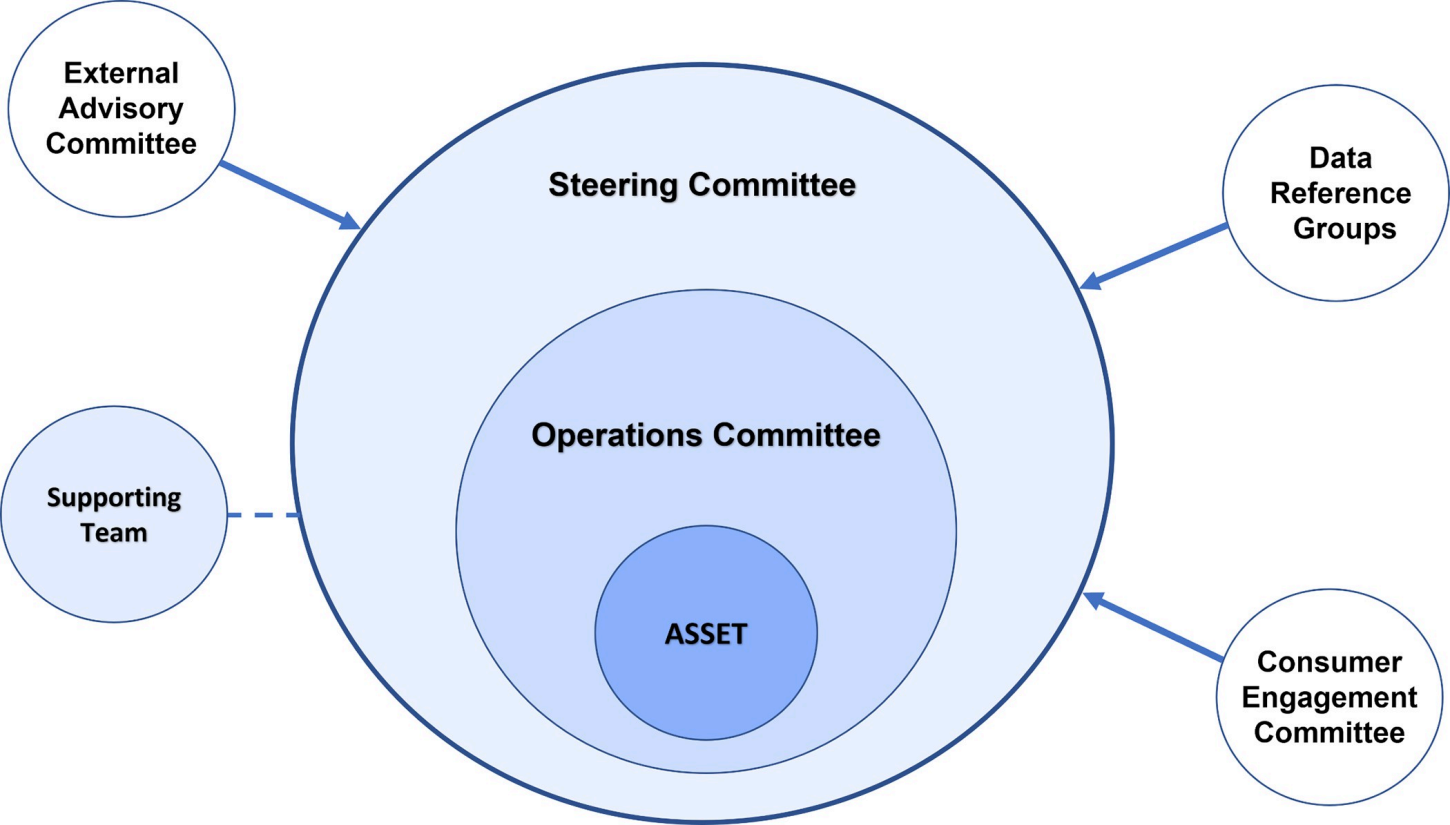
Governance framework of the ASSET data platform. Including Operations Committee, Steering Committee, External Advisory Committee, Data Reference Groups, Consumer Engagement Committee and Supporting Team.

ASSET’s data governance framework (Fig 4) will form the basis for management of the ASSET data platform. Decision making, oversight and accountability of the data platform will be the responsibility of the ASSET Steering Committee, in collaboration with the Operations Committee, External Advisory Committee, Consumer Engagement Committee and Data Reference Groups. The Operations Committee will be responsible for the day-to-day running of ASSET and related projects, including data linkage processes, approvals, storage, and data transfers. The Steering Committee will be responsible for advising and providing feedback on the research produced from ASSET and communicating findings and research progress to the External Advisory Committee, Consumer Engagement Committee and Data Reference Groups. The Steering Committee will uphold and be accountable for data security in line with Ethics and Data Custodians. The data platform will be held at the University of Sydney. However, the Steering Committee will ensure data availability to other organisations or persons with reasonable requests is provided under agreed conditions and pending any other approvals.

All groups will meet every six months to discuss ASSET’s strategic plan including research project proposals, study design, analysis plans, outputs and future direction. As data custodians, Data Reference Groups will provide updated data and additional datasets for biennial re-linkage and provide advice on analyses and findings produced. The Consumer Engagement Committee will engage consumers, provide input and represent consumer values and preferences in health service delivery, ensuring cultural inclusion, and assist in disseminating research findings through consumer channels. The External Advisory Committee will provide advice on the relevance and impact of the research on current health policies, ensure governance of the data platform is appropriate and adhered to and provide expert advice on ASSET’s strategic plan. Supporting teams will be supervised by the Steering Committee and aid in research and project management.

## Discussion

The New Zealand Ministry of Health acknowledges achieving health equity requires intersectional action to address social determinants of health and improve health service design and distribution [39]. Recognising and understanding where equity gaps occur in the system and identifying where priorities for investment and resources lie is outlined as a requirement for achieving health equity by the New Zealand Ministry of Health in 2019 [21]. One key focus of government strategy is to explore and understand issues of equity through data, analytics and multifaceted research, with particular desire for updated disaggregated and granular information on disadvantaged groups experiencing poorer outcomes [21]. Overall, using data to understand where issues of equity exist in the delivery of health services is reiterated as an important component to achieving health equity in New Zealand [21, 39]. The scope, design and aim of ASSET aligns with the New Zealand Ministry of Health’s strategy. Kidney failure treatment data is collected unilaterally in New Zealand and excludes granular information on social determinants of health. ASSET consists of individual linked data from across nine databases and registries, providing comprehensive information to support multi-faceted research with a focus on equity.

ASSET is enriched by overlapping expertise from investigators in research, renal and transplantation medicine, psychiatry, policy as well as consumers and Māori representatives. This collaboration will provide an integrated health services approach in investigating multiple objectives, with the aim of identifying inequities in the delivery of kidney failure health services. Using ASSET, we expect our preliminary findings will highlight which aspects of the health system could be improved to redress inequities and create a more equitable health care system for people needing treatment for kidney failure. This will build a foundation for future research to examine health service delivery deficits and develop targeted health delivery interventions to increase equity in kidney health service delivery.

We plan to disseminate our findings in peer-reviewed journal articles, scientific and medical conferences. Also, via consumers and health care professionals listed as investigators in the study who maintain direct contact with transplant recipients and donors in the New Zealand health care system. Findings will also be disseminated through partner organisations and consumer groups represented in our governance framework.

### Strengths and limitations

The strengths of our study include the establishment of a comprehensive linked data platform, which includes all people receiving dialysis, all people ever waitlisted or worked up for a living kidney transplant, all kidney transplant recipients including pre-emptive transplants and all living donors in New Zealand. The ASSET data linkage platform is a rich data resource and supports multiple enquiries in health services research. It also provides a more detailed view of individual health and health services use over time.

However, there are limitations. Administrative and health datasets that are not collected with a research focus may unintentionally exclude data that informs research. One such example is establishing the date of referral to the waitlist. The New Zealand Blood Service data collects other dates that can be used as a proxy (e.g. date of HLA typing), but the total length of time from referral to assessment for waitlisting will not be known. ASSET will not capture those with kidney failure who transitioned to supportive care and never received dialysis or commenced transplant assessment. Further, patients who did not receive dialysis and commenced transplant assessment, but subsequently did not undergo HLA typing will not be captured.

PRIMHD database often contains missing or incomplete mental illness diagnostic coding, therefore the pharmaceutical claims database will complement this database to determine mental illness diagnosis via medication therapy. Also, health registries may not capture all comorbidities, nor the severity of the condition listed for each individual. Data from the pharmaceutical claims database may provide medications that relate to co-morbidities, yet it is hard to ascertain direct therapeutic use as medications can be used for a range of different treatments. Also, the pharmaceutical claims database does not capture over the counter medication purchases, or medications dispensed by hospitals.

## Conclusion

ASSET is a rich comprehensive data linkage platform for kidney failure research in New Zealand. Our findings will support the design and implementation of health delivery interventions that will be most impactful in promoting equity in kidney transplantation in New Zealand.
